# Supplementary material for: Complexome profiling of the Chlamydomonas psb28 mutant reveals TEF5 as an early PSII assembly factor
Source: Plant Cell. 2025 Mar 17;37(6):koaf055. doi: 10.1093/plcell/koaf055 (PMC12164590; doi:10.1093/plcell/koaf055)
Supplement: koaf055_Supplementary_Data [file koaf055_supplementary_data.zip › psb28_interactive.zip!/plots/intensity/qualityPlots/peptides/RPL4_Cre09.g397697.t1.1.html]

### Cre09.g397697.t1.1

Trivial name: RPL4  
SeqCoverage [median]: 7.30  
Mass: 44.91 kDa  
  
 

| Sequence | MissedCleavage | Mass [Da] | wtIntensity | muIntensity | Score |
| --- | --- | --- | --- | --- | --- |
| KGPLVVFANDEGISK | 1 | 1572.85 | 0.00 | 434.02 | 47.53 |
| LINSDEIQSAVRPAK | 1 | 1639.89 | 1002.81 | 774.14 | 48.11 |
|  |  |  | 1002.8 | 1208.2 |  |
